# Case Report: CT diagnosis of thymic remnant cyst/thymopharyngeal duct cyst

**DOI:** 10.4103/0971-3026.57211

**Published:** 2009-11

**Authors:** Bipin V Daga, VA Chaudhary, VB Dhamangaokar

**Affiliations:** Department of Radiodiagnosis and Imaging Sciences, Dr. Vaishampaiyan Memorial Government Medical College, Solapur, Maharashtra, India

**Keywords:** CT, neck swelling, thymic remnant cyst

## Abstract

A 4-year-old boy presented with history of left anterolateral neck swelling since birth. He was clinically diagnosed to have a branchial cleft cyst. A CT scan revealed findings suggestive of a thymic remnant cyst. The lesion was excised and the diagnosis was confirmed by histopathology.

## Introduction

A thymic remnant cyst (TRC) is a rare lesion. The diagnosis cannot be established solely on the basis of the clinical findings. The main differential diagnosis is a second branchial cleft cyst and imaging is necessary for diagnosis. It not only permits a firm diagnosis to be made but also depicts the exact anatomical location, the relationship of the cyst to the contents of the carotid sheath, and the extent of the lesion.[1]

## Case Report

**Figure 1:**
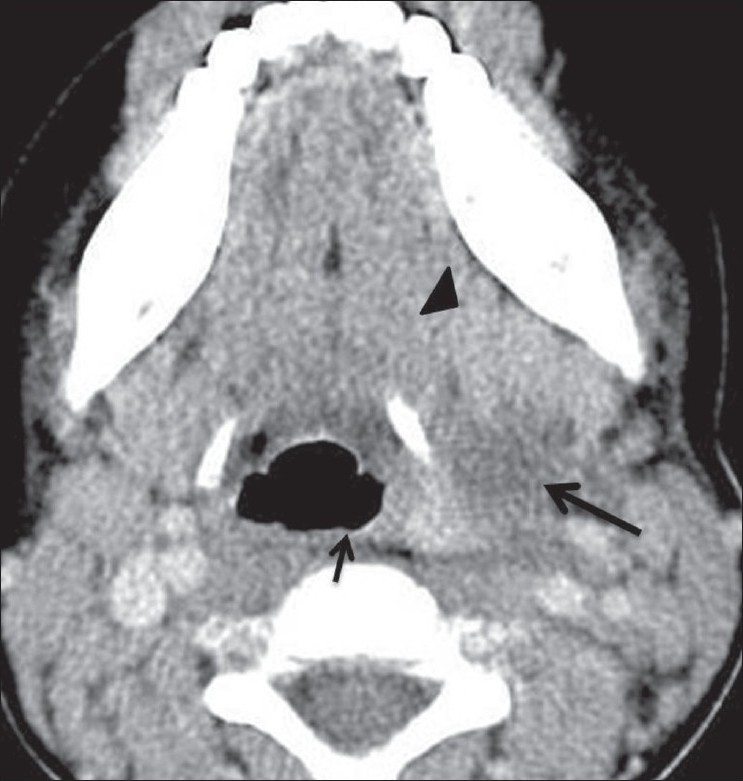
Axial CT scan of the neck shows the cranial (proximal) end of the lesion (arrow) and its relation to the left submandibular gland (arrowhead) and pyriform sinus (short arrow)

**Figure 2:**
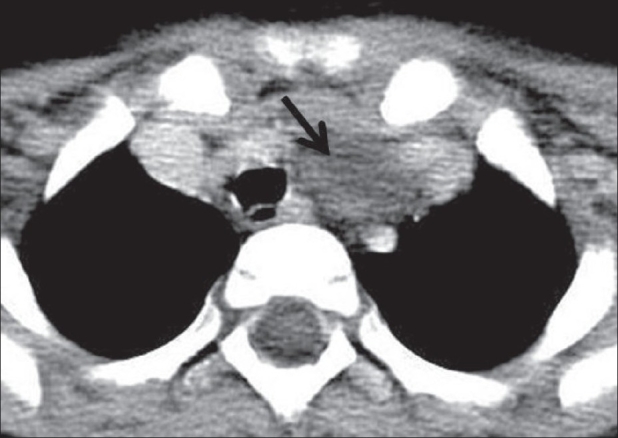
Axial CT scan of the neck shows the caudal (distal) end of the lesion (arrow), extending into the superior mediastinum, and its relation to the thymus

**Figure 3:**
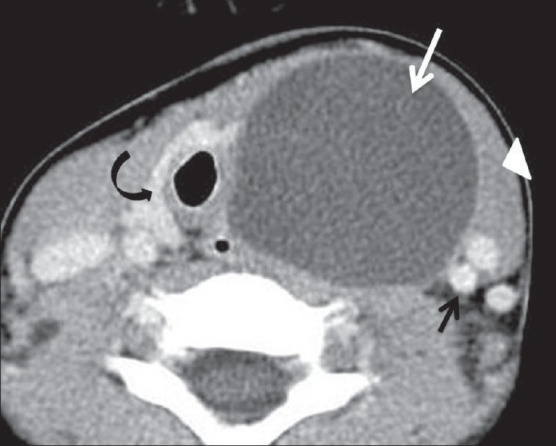
Axial CT scan of the neck shows the relationship of the lesion (arrow) with the sternocleidomastoid muscle (arrowhead), carotid vessels (short arrow), and the thyroid gland (curved arrow)

A 4-year-old boy presented with an asymptomatic, left-sided neck swelling that had been present since birth. He underwent a CT scan of the neck, which revealed a 6.7 × 4.3 × 3.3-cm, well-defined, thin-walled, cystic lesion on the left side, with CT attenuation values of 5–10 HU and subtle peripheral rim enhancement. The lesion was deep to the left sternocleidomastoid muscle and parallel to it. It extended cranially up to the level of the left pyriform sinus [Figure 1] and caudally into the superior mediastinum, up to the thymus [Figure 2]. No obvious connection with the thymus was noted [Figure 3]. The lesion, along its entire extent, was located medial to the carotid vessels, displacing them laterally [Figure 3]. The lesion also displaced the trachea and the thyroid gland to the right. There was subtle indentation of the left lateral wall of the trachea [Figure 3]. No obvious communication with the pharynx, larynx, or thyroid gland was noted [Figure 2]. Considering the anatomical location of the lesion, its relation to the sternocleidomastoid and the carotid vessels, and its caudal extent into the superior mediastinum, a diagnosis of TRC was made. The patient was operated with no significant postoperative event and the diagnosis was confirmed on histopathological examination.

## Discussion

The thymus develops from the third pharyngeal pouch. During development, it traverses the neck to its final position in the anterosuperior mediastinum. Thymic remnants are therefore likely to be found in the neck, often presenting as neck masses.[2,3]

One type of thymic remnant is the TRC, a remnant of one of the paired tracts of embryological thymic descent. TRCs are rare lesions and are also referred to as thymopharyngeal cysts because they presumably follow the course of resorbed thymopharyngeal ducts.[4–6]

About two-thirds of the TRCs occur in the first decade of life and the remaining in the second and the third decades. The majority of these cysts are located on the left side of the neck.[2]

Although they can present anywhere in the neck from the angle of the mandible to the sternum, most TRCs are present in the lower neck as slowly enlarging masses. They are usually oriented along the long axis of the sternocleidomastoid muscle. They may be completely isolated from the normal thymic tissue or may be attached to the thymus by a fibrous band.[3]

On imaging, a TRC usually appears as a solitary, off-midline (usually left-sided) cyst, situated low in the anterior neck, either adjacent to the lower part of the pyriform sinus or just caudal to the thyroid gland. On USG, TRC appear as an anechoic neck mass with thin, distinct walls, extending up through the thoracic inlet behind the thyroid.[7] A CT scan shows the cyst wall to be uniformly smooth and delicate. The CT attenuation values of the contents of the cyst range from 10 to 25 HU. Usually, there is no demonstrable connection to the thymus in the anterior mediastinum. After contrast administration, TRCs usually show a peripheral thin rim of enhancement. On MRI, TRCs are seen as T2-hyperintense cervicomediastinal masses typically situated between the internal jugular vein and the carotid vessels.[4–6]

The differential diagnosis of TRC includes conditions such as thyroglossal cyst, malformation of the lymphatic system, cystic neuroblastoma, external laryngocele, vallecular cyst, and lymphadenopathy.[5] The most important, however, is the second branchial cleft cyst. It is possible to definitely differentiate between a TRC and a second branchial cyst with cross-sectional imaging. Whereas a second branchial cyst is seen to pass between the internal and the external carotid arteries to terminate at the base of the tonsils, the TRC typically passes behind the fork of the carotid to end in the pyriform sinus. Also, in general, TRCs are relatively longer, sometimes extending downwards as far as the anterosuperior compartment of the mediastinum. In contrast, mediastinal extension does not occur with branchial cleft anomalies. As a general rule, when the neck mass is situated close to the lower pole of the thyroid, one must consider a thymic origin.

To summarize, we conclude that although the TRC is a rare lesion, it must be considered in the differential diagnosis of neck masses. CT scan is helpful in delineating the exact anatomical location, the relationship to the contents of the carotid sheath, and the extent of this uncommon neck mass.
